# Bis[*N*,*N*-dimethyl-1-(10*H*-pyrido[3,2-*b*][1,4]benzothia­zin-10-yl)propan-2-aminium] tetrakis­(thio­cyanato-κ*N*)cobaltate(II)

**DOI:** 10.1107/S1600536810021367

**Published:** 2010-06-16

**Authors:** H. K. Arunkashi, S. Jeyaseelan, Suresh Babu Vepuri, H. D. Revanasiddappa, H. C. Devarajegowda

**Affiliations:** a Department of Physics, Yuvaraja’s College (Constituent College), University of Mysore, Mysore 570 005, Karnataka, India; b GITAM Institute of Pharmacy, GITAM University, Visakhapatnam 530 045, Andhrapradesh, India; c Department of Studies in Chemistry, Manasagangotri, University of Mysore, Mysore 570 006, Karnataka, India

## Abstract

The asymmetric unit of the title salt, (C_16_H_20_N_3_S)_2_[Co(NCS)_4_], comprises one monovalent isothio­pendylium cation and one-half of a divalent thio­cyanatocobaltate(II) anion (2 symmetry). The central thia­zine ring of the cation is slightly twisted in a boat-like fashion, with r.m.s. deviations from the mean plane of 0.272 (1) and 0.2852 (8) Å for the N and S atoms. The mol­ecular structure of the cation is stabilized by an intra­molecular N—H⋯N hydrogen bond. Within the complex anion, the Co^II^ atom is tetra­hedrally surrounded by four N atoms of the thio­cyanate ligands. π–π stacking, with a distance of 3.7615 (10) Å between the centroids of benzene and pyridine rings, helps to consolidate the packing.

## Related literature

For general background to isothipendyl, cobalt(II) and thio­cyanate compounds, see: Kinnamon *et al.* (1994 ▶); Moreau *et al.* (1995 ▶); Scott *et al.* (1990 ▶); Hudson *et al.* (2005 ▶). For a related structure, see: Shi *et al.* (2005 ▶). 
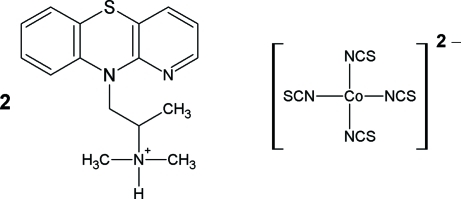


## Experimental

### 

#### Crystal data

(C_16_H_20_N_3_S)_2_[Co(NCS)_4_]
*M* *_r_* = 864.07
Monoclinic, 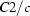

*a* = 25.2420 (4) Å
*b* = 11.4357 (2) Å
*c* = 14.5939 (2) Å
β = 98.557 (1)°
*V* = 4165.78 (11) Å^3^
*Z* = 4
Mo *K*α radiation
μ = 0.75 mm^−1^
*T* = 295 K
0.22 × 0.15 × 0.12 mm

#### Data collection

Bruker APEXII CCD area-detector diffractometer
Absorption correction: ψ scan (*SADABS*; Sheldrick, 2004 ▶) *T*_min_ = 0.852, *T*_max_ = 0.915
48347 measured reflections
6498 independent reflections
4566 reflections with *I* > 2σ(*I*)
*R*_int_ = 0.032

#### Refinement

*R*[*F* ^2^ > 2σ(*F* ^2^)] = 0.038
*wR*(*F* ^2^) = 0.114
*S* = 1.01
6498 reflections
243 parameters
H-atom parameters constrained
Δρ_max_ = 0.30 e Å^−3^
Δρ_min_ = −0.37 e Å^−3^

### 

Data collection: *APEX2* (Bruker, 2008 ▶); cell refinement: *SAINT* (Bruker, 2008 ▶); data reduction: *SAINT*; program(s) used to solve structure: *SHELXS97* (Sheldrick, 2008 ▶); program(s) used to refine structure: *SHELXL97* (Sheldrick, 2008 ▶); molecular graphics: *ORTEP-3* (Farrugia, 1997 ▶); software used to prepare material for publication: *SHELXL97*.

## Supplementary Material

Crystal structure: contains datablocks I, global. DOI: 10.1107/S1600536810021367/wm2356sup1.cif

Structure factors: contains datablocks I. DOI: 10.1107/S1600536810021367/wm2356Isup2.hkl

Additional supplementary materials:  crystallographic information; 3D view; checkCIF report

## Figures and Tables

**Table:** 

Co1—N4	1.9411 (19)
Co1—N5	1.9626 (16)

**Table:** 

N4^i^—Co1—N4	113.17 (13)
N4—Co1—N5	108.95 (7)
N4—Co1—N5^i^	110.33 (8)
N5—Co1—N5^i^	104.78 (9)

Symmetry code: (i) 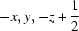.

**Table 2:** Hydrogen-bond geometry (Å, °)

*D*—H⋯*A*	*D*—H	H⋯*A*	*D*⋯*A*	*D*—H⋯*A*
N3—H3*A*⋯N1	0.91	1.91	2.7494 (18)	152

**Fig. 1.**
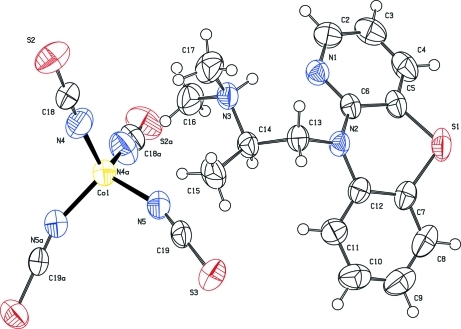
The molecular structure of the title compound. Displacement ellipsoids are drawn at the 50% probability level. Symmetry code a) -x, y, -z+1/2.

**Fig. 2.**
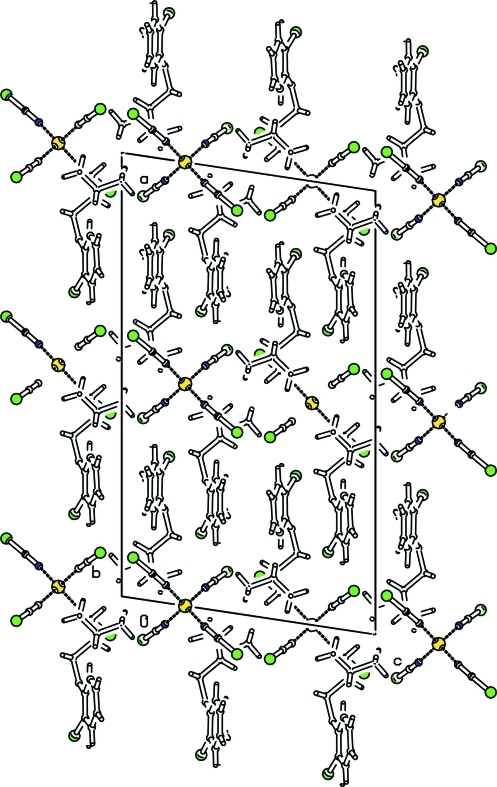
Packing of the molecules as viewed down [010].

## supplementary crystallographic information

### Comment

The molecular structure of isothipendyl, C_16_H_19_N_3_S, is close to that of phenothiazines. Isothipendyl is an antihistamine used in the treatment of allergies. It was also found to be active against parasites causing filariasis (Kinnamon *et al.*, 1994). Photobiological properties of isothipendyl were also investigated and found to have ultraviolet B (UVB) protective activity (Moreau *et al.*, 1995). Studies also suggest that cobalt and thiocyanates play some role in phototoxicity and in the development of conjugates for photoimmunotherapy (Scott *et al.*, 1990; Hudson *et al.*, 2005). These outcomes arouse our interest and we prepared the title salt [(C_16_H_20_N_3_S)_2_{Co(NCS)_4_}], (I), for structural characterisation.

In the structure of (I), the Co atom of the anion is situated on a twofold rotation axis and is coordinated by four N atoms from four thiocyanate groups in a slightly distorted tetrahedral geometry (Fig. 1, Table 1). The bond lengths and bond angles of the cobaltate(II) anion are in good agreement with related structures (Shi *et al.*, 2005).

Within the cation the dihedral angles between the benzene and the thiazine rings and between the pyridine and the thiazine rings are 15.73 (8)° and 14.77 (8)°, respectively. The central thiazine ring is slightly twisted as boat like. The deviation of the N and S atoms from the mean plane of the thiazine ring was found to be 0.272 (1) and 0.2852 (8) Å, respectively. The structure displays an intramolecular hydrogen bonding interaction between N3–H3A···N1 (Fig. 2 & Table 2).

There are significant π—π stacking interactions between the pyridine and benzene rings; the relevant distances are *Cg*2—*Cg*3^i^ = 3.7615 (10) Å and *Cg*2—3^i^_perp_ = 3.6975 (7) Å, and *Cg*3—*Cg*2^ii^ = 3.7614 (10) Å and *Cg*3—2^ii^_perp_ = 3.6820 (7) Å [symmetry codes: (i) 1/2 - *x*,1/2 + *y*,3/2 - *z*;(ii) 1/2 - *x*, -1/2 + *y*,3/2 - *z*; *Cg*2 and *Cg*3 are the centroids of the N1/C2–C6 and C7–C12 rings, respectively; *CgI*—*J*_perp_ is the perpendicular distance from *CgI* to ring *J*]. In addition, there are weak intermolecular C2—H2···S2 and C16—H16A···S2 interactions with H···S distances of 2.886 (10) and 2.919 (10) Å and D—H···A angles of 154.54 (12)° and 138.40 (14)°, respectively.

In the crystal structure, molecules stack along [010] (Fig. 3).

### Experimental

The cobalt(II) salt was prepared by a single step method. Isothipendyl in ethanol (5 mmol) was slowly mixed with an ethanolic solution (5 mmol) of Co(SCN)_2_^.^2H_2_O. The mixture was kept at room temperature for 30 min and warmed on a water bath (343–353 K) for 1 h. Green crystals suitable for X-ray diffraction were obtained by slow evaporation of the solvent (M.P. 453 K; Yield 79%).

### Refinement

All H atoms were positioned at calculated positions with N—H = 0.91Å, C—H = 0.93 Å for aromatic H atoms, 0.97 Å for methylene H atoms and 0.96Å for methyl H atoms. H atoms were refined using a riding model with *U*_iso_(H) = 1.5*U*_eq_(C) for methyl H and *U*_iso_(H) = 1.2*U*_eq_(X) for other atoms (X = N, C).

### Crystal data

**Table:**

(C_16_H_20_N_3_S)_2_[Co(NCS)_4_]	*F*(000) = 1796
*M**_r_* = 864.07	*D*_x_ = 1.378 Mg m^−^^3^
Monoclinic, *C*2/*c*	Melting point: 453 K
Hall symbol: -C 2yc	Mo *K*α radiation, λ = 0.71073 Å
*a* = 25.2420 (4) Å	Cell parameters from 6498 reflections
*b* = 11.4357 (2) Å	θ = 1.6–30.8°
*c* = 14.5939 (2) Å	µ = 0.75 mm^−^^1^
β = 98.557 (1)°	*T* = 295 K
*V* = 4165.78 (11) Å^3^	Plate, green
*Z* = 4	0.22 × 0.15 × 0.12 mm

### Data collection

**Table:**

Bruker APEXII CCD area-detector diffractometer	6498 independent reflections
Radiation source: fine-focus sealed tube	4566 reflections with *I* > 2σ(*I*)
graphite	*R*_int_ = 0.032
ω and φ scans	θ_max_ = 30.8°, θ_min_ = 1.6°
Absorption correction: ψ scan (*SADABS*; Sheldrick, 2004)	*h* = −36→36
*T*_min_ = 0.852, *T*_max_ = 0.915	*k* = −16→16
48347 measured reflections	*l* = −20→20

### Refinement

**Table:**

Refinement on *F*^2^	Primary atom site location: structure-invariant direct methods
Least-squares matrix: full	Secondary atom site location: difference Fourier map
*R*[*F*^2^ > 2σ(*F*^2^)] = 0.038	Hydrogen site location: inferred from neighbouring sites
*wR*(*F*^2^) = 0.114	H-atom parameters constrained
*S* = 1.01	*w* = 1/[σ^2^(*F*_o_^2^) + (0.0516*P*)^2^ + 2.3179*P*] where *P* = (*F*_o_^2^ + 2*F*_c_^2^)/3
6498 reflections	(Δ/σ)_max_ < 0.001
243 parameters	Δρ_max_ = 0.30 e Å^−^^3^
0 restraints	Δρ_min_ = −0.37 e Å^−^^3^

### Special details

**Table:**

Geometry. All e.s.d.'s (except the e.s.d. in the dihedral angle between two l.s. planes) are estimated using the full covariance matrix. The cell e.s.d.'s are taken into account individually in the estimation of e.s.d.'s in distances, angles and torsion angles; correlations between e.s.d.'s in cell parameters are only used when they are defined by crystal symmetry. An approximate (isotropic) treatment of cell e.s.d.'s is used for estimating e.s.d.'s involving l.s. planes.
Refinement. Refinement of *F*^2^ against ALL reflections. The weighted *R*-factor *wR* and goodness of fit *S* are based on *F*^2^, conventional *R*-factors *R* are based on *F*, with *F* set to zero for negative *F*^2^. The threshold expression of *F*^2^ > σ(*F*^2^) is used only for calculating *R*-factors(gt) *etc*. and is not relevant to the choice of reflections for refinement. *R*-factors based on *F*^2^ are statistically about twice as large as those based on *F*, and *R*- factors based on ALL data will be even larger.

### Fractional atomic coordinates and isotropic or equivalent isotropic displacement parameters (Å^2^)

**Table:**

	*x*	*y*	*z*	*U*_iso_*/*U*_eq_	
Co1	0.0000	0.18800 (3)	0.2500	0.05395 (11)	
S1	0.169598 (17)	0.36282 (5)	0.31347 (4)	0.06906 (15)	
S2	0.09133 (3)	0.39599 (6)	0.04513 (5)	0.0913 (2)	
S3	−0.09097 (2)	−0.11877 (5)	0.08464 (4)	0.06810 (15)	
N1	0.30192 (5)	0.17216 (12)	0.37667 (10)	0.0479 (3)	
N2	0.29026 (5)	0.36667 (11)	0.33141 (9)	0.0428 (3)	
N3	0.40850 (5)	0.23282 (13)	0.40260 (9)	0.0503 (3)	
H3A	0.3781	0.1888	0.3953	0.060*	
N4	0.04020 (8)	0.28147 (18)	0.17318 (14)	0.0771 (5)	
N5	−0.04823 (6)	0.08326 (15)	0.17017 (11)	0.0595 (4)	
C2	0.28169 (8)	0.06803 (16)	0.39813 (13)	0.0589 (4)	
H2	0.3049	0.0049	0.4101	0.071*	
C3	0.22864 (9)	0.05145 (18)	0.40307 (14)	0.0663 (5)	
H3	0.2161	−0.0205	0.4204	0.080*	
C4	0.19412 (7)	0.14419 (19)	0.38174 (14)	0.0621 (5)	
H4	0.1578	0.1352	0.3848	0.074*	
C5	0.21316 (6)	0.24990 (16)	0.35591 (11)	0.0487 (4)	
C6	0.26864 (5)	0.26126 (14)	0.35550 (10)	0.0413 (3)	
C7	0.21115 (6)	0.48282 (17)	0.34832 (11)	0.0506 (4)	
C8	0.18804 (8)	0.5880 (2)	0.36902 (13)	0.0653 (5)	
H8	0.1513	0.5917	0.3700	0.078*	
C9	0.21887 (10)	0.6863 (2)	0.38804 (14)	0.0729 (6)	
H9	0.2030	0.7567	0.4007	0.087*	
C10	0.27306 (10)	0.68031 (18)	0.38826 (14)	0.0693 (5)	
H10	0.2939	0.7472	0.4000	0.083*	
C11	0.29708 (8)	0.57504 (16)	0.37113 (12)	0.0562 (4)	
H11	0.3341	0.5715	0.3736	0.067*	
C12	0.26658 (6)	0.47499 (14)	0.35030 (10)	0.0443 (3)	
C13	0.34445 (6)	0.36661 (15)	0.30759 (10)	0.0440 (3)	
H13A	0.3491	0.4375	0.2732	0.053*	
H13B	0.3476	0.3011	0.2666	0.053*	
C14	0.39047 (6)	0.35898 (15)	0.38957 (11)	0.0459 (3)	
H14	0.3763	0.3831	0.4456	0.055*	
C15	0.43718 (7)	0.4390 (2)	0.37864 (16)	0.0689 (5)	
H15A	0.4659	0.4254	0.4285	0.103*	
H15B	0.4258	0.5190	0.3799	0.103*	
H15C	0.4494	0.4230	0.3206	0.103*	
C16	0.43609 (9)	0.2099 (2)	0.49819 (14)	0.0752 (6)	
H16A	0.4490	0.1308	0.5024	0.113*	
H16B	0.4114	0.2211	0.5414	0.113*	
H16C	0.4657	0.2628	0.5125	0.113*	
C17	0.44122 (9)	0.1896 (2)	0.33280 (17)	0.0764 (6)	
H17A	0.4771	0.2191	0.3474	0.115*	
H17B	0.4258	0.2162	0.2723	0.115*	
H17C	0.4419	0.1057	0.3337	0.115*	
C18	0.06195 (8)	0.32922 (17)	0.12044 (15)	0.0622 (5)	
C19	−0.06629 (6)	−0.00127 (16)	0.13474 (12)	0.0492 (4)	

### Atomic displacement parameters (Å^2^)

**Table:**

	*U*^11^	*U*^22^	*U*^33^	*U*^12^	*U*^13^	*U*^23^
Co1	0.04465 (17)	0.0540 (2)	0.0604 (2)	0.000	−0.00135 (13)	0.000
S1	0.03187 (19)	0.0899 (4)	0.0813 (3)	0.0035 (2)	−0.00519 (19)	0.0002 (3)
S2	0.0961 (5)	0.0837 (4)	0.0981 (5)	−0.0315 (4)	0.0272 (4)	0.0016 (3)
S3	0.0596 (3)	0.0733 (3)	0.0717 (3)	−0.0168 (2)	0.0107 (2)	−0.0148 (2)
N1	0.0407 (6)	0.0498 (7)	0.0536 (7)	−0.0035 (5)	0.0078 (5)	0.0019 (6)
N2	0.0304 (5)	0.0487 (7)	0.0503 (7)	−0.0008 (5)	0.0087 (5)	0.0030 (6)
N3	0.0354 (6)	0.0617 (8)	0.0528 (7)	0.0017 (6)	0.0038 (5)	−0.0058 (6)
N4	0.0690 (11)	0.0815 (12)	0.0766 (11)	−0.0245 (9)	−0.0027 (9)	0.0100 (10)
N5	0.0439 (7)	0.0615 (9)	0.0701 (9)	−0.0001 (7)	−0.0014 (6)	−0.0029 (8)
C2	0.0628 (10)	0.0498 (9)	0.0644 (10)	−0.0054 (8)	0.0108 (8)	0.0037 (8)
C3	0.0728 (12)	0.0586 (11)	0.0709 (12)	−0.0233 (10)	0.0218 (10)	−0.0009 (9)
C4	0.0458 (8)	0.0757 (12)	0.0674 (11)	−0.0228 (9)	0.0172 (8)	−0.0099 (10)
C5	0.0332 (6)	0.0644 (10)	0.0484 (8)	−0.0073 (7)	0.0055 (6)	−0.0045 (7)
C6	0.0326 (6)	0.0525 (8)	0.0389 (7)	−0.0047 (6)	0.0054 (5)	−0.0021 (6)
C7	0.0433 (7)	0.0657 (10)	0.0418 (7)	0.0115 (7)	0.0031 (6)	0.0061 (7)
C8	0.0597 (10)	0.0838 (14)	0.0521 (9)	0.0296 (10)	0.0071 (8)	0.0072 (9)
C9	0.0941 (16)	0.0655 (13)	0.0572 (11)	0.0290 (12)	0.0056 (10)	0.0009 (9)
C10	0.0937 (16)	0.0514 (10)	0.0614 (11)	0.0053 (10)	0.0066 (10)	0.0023 (9)
C11	0.0585 (10)	0.0530 (10)	0.0565 (9)	−0.0007 (8)	0.0066 (8)	0.0047 (8)
C12	0.0431 (7)	0.0520 (9)	0.0375 (7)	0.0056 (6)	0.0046 (5)	0.0057 (6)
C13	0.0340 (6)	0.0550 (9)	0.0448 (7)	−0.0025 (6)	0.0115 (5)	0.0029 (6)
C14	0.0322 (6)	0.0567 (9)	0.0495 (8)	−0.0044 (6)	0.0085 (6)	−0.0045 (7)
C15	0.0421 (8)	0.0783 (13)	0.0871 (14)	−0.0189 (9)	0.0116 (9)	−0.0023 (11)
C16	0.0714 (13)	0.0858 (15)	0.0613 (11)	0.0179 (11)	−0.0133 (10)	−0.0035 (10)
C17	0.0584 (11)	0.0925 (16)	0.0811 (14)	0.0187 (11)	0.0194 (10)	−0.0162 (12)
C18	0.0510 (9)	0.0556 (10)	0.0757 (12)	−0.0121 (8)	−0.0049 (9)	−0.0033 (9)
C19	0.0310 (6)	0.0614 (10)	0.0546 (9)	0.0033 (6)	0.0048 (6)	0.0030 (8)

### Geometric parameters (Å, °)

**Table:**

Co1—N4^i^	1.9411 (19)	C5—C6	1.4073 (19)
Co1—N4	1.9411 (19)	C7—C8	1.389 (3)
Co1—N5	1.9626 (16)	C7—C12	1.398 (2)
Co1—N5^i^	1.9626 (16)	C8—C9	1.372 (3)
S1—C5	1.7485 (18)	C8—H8	0.9300
S1—C7	1.756 (2)	C9—C10	1.369 (3)
S2—C18	1.607 (2)	C9—H9	0.9300
S3—C19	1.6102 (19)	C10—C11	1.387 (3)
N1—C6	1.328 (2)	C10—H10	0.9300
N1—C2	1.351 (2)	C11—C12	1.387 (2)
N2—C6	1.390 (2)	C11—H11	0.9300
N2—C12	1.420 (2)	C13—C14	1.541 (2)
N2—C13	1.4608 (18)	C13—H13A	0.9700
N3—C16	1.487 (2)	C13—H13B	0.9700
N3—C17	1.488 (2)	C14—C15	1.519 (2)
N3—C14	1.516 (2)	C14—H14	0.9800
N3—H3A	0.9100	C15—H15A	0.9600
N4—C18	1.148 (3)	C15—H15B	0.9600
N5—C19	1.157 (2)	C15—H15C	0.9600
C2—C3	1.365 (3)	C16—H16A	0.9600
C2—H2	0.9300	C16—H16B	0.9600
C3—C4	1.378 (3)	C16—H16C	0.9600
C3—H3	0.9300	C17—H17A	0.9600
C4—C5	1.374 (3)	C17—H17B	0.9600
C4—H4	0.9300	C17—H17C	0.9600
			
N4^i^—Co1—N4	113.17 (13)	C8—C9—H9	120.1
N4^i^—Co1—N5	110.33 (8)	C9—C10—C11	120.4 (2)
N4—Co1—N5	108.95 (7)	C9—C10—H10	119.8
N4^i^—Co1—N5^i^	108.95 (7)	C11—C10—H10	119.8
N4—Co1—N5^i^	110.33 (8)	C10—C11—C12	120.80 (19)
N5—Co1—N5^i^	104.78 (9)	C10—C11—H11	119.6
C5—S1—C7	99.06 (7)	C12—C11—H11	119.6
C6—N1—C2	118.85 (14)	C11—C12—C7	118.22 (16)
C6—N2—C12	121.00 (12)	C11—C12—N2	121.74 (14)
C6—N2—C13	118.47 (12)	C7—C12—N2	120.05 (15)
C12—N2—C13	118.87 (13)	N2—C13—C14	116.08 (12)
C16—N3—C17	110.70 (16)	N2—C13—H13A	108.3
C16—N3—C14	112.07 (14)	C14—C13—H13A	108.3
C17—N3—C14	114.63 (15)	N2—C13—H13B	108.3
C16—N3—H3A	106.3	C14—C13—H13B	108.3
C17—N3—H3A	106.3	H13A—C13—H13B	107.4
C14—N3—H3A	106.3	N3—C14—C15	111.28 (14)
C18—N4—Co1	172.97 (18)	N3—C14—C13	109.19 (13)
C19—N5—Co1	160.60 (14)	C15—C14—C13	113.05 (14)
N1—C2—C3	122.85 (19)	N3—C14—H14	107.7
N1—C2—H2	118.6	C15—C14—H14	107.7
C3—C2—H2	118.6	C13—C14—H14	107.7
C2—C3—C4	118.25 (18)	C14—C15—H15A	109.5
C2—C3—H3	120.9	C14—C15—H15B	109.5
C4—C3—H3	120.9	H15A—C15—H15B	109.5
C5—C4—C3	120.25 (16)	C14—C15—H15C	109.5
C5—C4—H4	119.9	H15A—C15—H15C	109.5
C3—C4—H4	119.9	H15B—C15—H15C	109.5
C4—C5—C6	118.11 (17)	N3—C16—H16A	109.5
C4—C5—S1	121.32 (13)	N3—C16—H16B	109.5
C6—C5—S1	120.26 (13)	H16A—C16—H16B	109.5
N1—C6—N2	117.76 (12)	N3—C16—H16C	109.5
N1—C6—C5	121.59 (15)	H16A—C16—H16C	109.5
N2—C6—C5	120.64 (14)	H16B—C16—H16C	109.5
C8—C7—C12	120.16 (18)	N3—C17—H17A	109.5
C8—C7—S1	119.17 (14)	N3—C17—H17B	109.5
C12—C7—S1	120.58 (13)	H17A—C17—H17B	109.5
C9—C8—C7	120.63 (19)	N3—C17—H17C	109.5
C9—C8—H8	119.7	H17A—C17—H17C	109.5
C7—C8—H8	119.7	H17B—C17—H17C	109.5
C10—C9—C8	119.72 (19)	N4—C18—S2	178.86 (19)
C10—C9—H9	120.1	N5—C19—S3	179.38 (17)
			
N4^i^—Co1—N5—C19	−126.5 (5)	S1—C7—C8—C9	−173.88 (15)
N4—Co1—N5—C19	108.7 (5)	C7—C8—C9—C10	−1.2 (3)
N5^i^—Co1—N5—C19	−9.4 (4)	C8—C9—C10—C11	−1.2 (3)
C6—N1—C2—C3	−3.0 (3)	C9—C10—C11—C12	2.3 (3)
N1—C2—C3—C4	2.5 (3)	C10—C11—C12—C7	−0.9 (3)
C2—C3—C4—C5	0.3 (3)	C10—C11—C12—N2	179.22 (16)
C3—C4—C5—C6	−2.4 (3)	C8—C7—C12—C11	−1.5 (2)
C3—C4—C5—S1	171.23 (15)	S1—C7—C12—C11	174.88 (12)
C7—S1—C5—C4	151.93 (15)	C8—C7—C12—N2	178.41 (14)
C7—S1—C5—C6	−34.59 (15)	S1—C7—C12—N2	−5.2 (2)
C2—N1—C6—N2	−178.41 (15)	C6—N2—C12—C11	147.52 (15)
C2—N1—C6—C5	0.7 (2)	C13—N2—C12—C11	−17.5 (2)
C12—N2—C6—N1	−149.64 (14)	C6—N2—C12—C7	−32.4 (2)
C13—N2—C6—N1	15.5 (2)	C13—N2—C12—C7	162.56 (14)
C12—N2—C6—C5	31.2 (2)	C6—N2—C13—C14	−77.11 (18)
C13—N2—C6—C5	−163.65 (14)	C12—N2—C13—C14	88.31 (17)
C4—C5—C6—N1	1.9 (2)	C16—N3—C14—C15	77.02 (19)
S1—C5—C6—N1	−171.75 (12)	C17—N3—C14—C15	−50.2 (2)
C4—C5—C6—N2	−178.98 (15)	C16—N3—C14—C13	−157.49 (15)
S1—C5—C6—N2	7.3 (2)	C17—N3—C14—C13	75.24 (17)
C5—S1—C7—C8	−150.12 (14)	N2—C13—C14—N3	96.79 (16)
C5—S1—C7—C12	33.45 (14)	N2—C13—C14—C15	−138.75 (16)
C12—C7—C8—C9	2.6 (3)		

Symmetry codes: (i) −*x*, *y*, −*z*+1/2.

### Hydrogen-bond geometry (Å, °)

**Table:**

*D*—H···*A*	*D*—H	H···*A*	*D*···*A*	*D*—H···*A*
N3—H3A···N1	0.91	1.91	2.7494 (18)	152
